# Reply to the letter : To consider the exercise density in the dose–response relationship: the idea is promising, the operationalization tricky!

**DOI:** 10.1007/s00421-025-06003-w

**Published:** 2025-11-12

**Authors:** Fabian Herold, Liye Zou, Paula Theobald, Patrick Manser, Ryan S. Falck, Qian Yu, Teresa Liu-Ambrose, Arthur F. Kramer, Kirk I. Erickson, Boris Cheval, Yanxia Chen, Matthew Heath, Zhihao Zhang, Toru Ishihara, Keita Kamijo, Soichi Ando, Joseph T. Costello, Mats Hallgren, David Moreau, Vahid Farrahi, David A. Raichlen, Emmanuel Stamatakis, Michael J. Wheeler, Neville Owen, Sebastian Ludyga, Henning Budde, Thomas Gronwald

**Affiliations:** 1 Department of Physiology, HMU Health and Medical University Erfurt, Erfurt, Thuringia Germany; 2 Body-Brain-Mind Laboratory, Shenzhen University, Shenzhen, China; 3 Research Group Degenerative and Chronic Diseases, Movement, Faculty of Health Sciences Brandenburg, University of Potsdam, Potsdam, Germany; 4 Division of Physiotherapy, Department of Neurobiology, Care Sciences and Society, Karolinska Institute, Stockholm, Sweden; 5 School of Biomedical Engineering, The University of British Columbia, Vancouver, BC Canada; 6 Aging, Mobility, and Cognitive Health Laboratory, Department of Physical Therapy, The University of British Columbia, Vancouver, BC Canada; 7 Djavad Mowafaghian Centre for Brain Health, Vancouver Coastal Health Research Institute, Vancouver, BC Canada; 8 Centre for Aging Solutions for Mobility, Activity, Rehabilitation and Technology (SMART) at Vancouver Coastal Health, Vancouver Coastal Health Research Institute, Vancouver, BC Canada; 9 Beckman Institute for Advanced Science and Technology, University of Illinois at Urbana-Champaign, Urbana, IL USA; 10 Department of Neuroscience, AdventHealth Research Institute, AdventHealth, Orlando, FL USA; 11 Department of Psychology, University of Pittsburgh, Pittsburgh, PA USA; 12 Center for the Neural Basis of Cognition, University of Pittsburgh, Pittsburgh, PA USA; 13 Department of Sport Sciences and Physical Education, Ecole Normale Supérieure Rennes, Bruz, France; 14 Laboratory VIPS2, University of Rennes, Rennes, France; 15 School of Kinesiology, Faculty of Health Sciences, University of Western Ontario, London, ON N6A 3K7 Canada; 16 Canadian Centre for Activity and Aging, University of Western Ontario, London, ON N6A 3K7 Canada; 17 Graduate Program in Neuroscience, University of Western Ontario, London, ON N6A 3K7 Canada; 18 Graduate School of Human Development and Environment, Kobe University, Kobe, Japan; 19 Faculty of Liberal Arts and Sciences, Chukyo University, Nagoya, Japan; 20 Graduate School of Informatics and Engineering, The University of Electro-Communications, Tokyo, Japan; 21 Extreme Environments Laboratory, School of Psychology, Sport and Health Sciences, University of Portsmouth, Portsmouth, UK; 22 Epidemiology of Psychiatric Conditions, Substance Use and Social Environment (EPiCSS), Department of Public Health Sciences, Karolinska Institute, Solna, Sweden; 23 Institute for Physical Activity and Nutrition (IPAN), School of Exercise and Nutrition Sciences, Deakin University, Geelong, Australia; 24 School of Psychology and Centre for Brain Research, University of Auckland, Auckland, New Zealand; 25 Institute for Sport and Sport Science, TU Dortmund University, Dortmund, Germany; 26 Human and Evolutionary Biology Section, Department of Biological Sciences, University of Southern California, Los Angeles, CA 90089 USA; 27 Department of Anthropology, University of Southern California, Los Angeles, CA 90089 USA; 28 Mackenzie Wearables Research Hub, Charles Perkins Centre, University of Sydney, Sydney, NSW Australia; 29 School of Health Sciences, Faculty of Medicine and Health, University of Sydney, Sydney, NSW Australia; 30 Physical Activity Laboratory, Baker Heart & Diabetes Institute, Melbourne, VIC Australia; 31 Centre for Urban Transitions, Swinburne University of Technology, Melbourne, VIC Australia; 32 Department of Sport, Exercise and Health, University of Basel, Basel, Switzerland; 33 Institute for Systems Medicine (ISM), MSH Medical School Hamburg University of Applied Sciences and Medical University Hamburg, Hamburg, Germany; 34 Institute of Interdisciplinary Exercise Science and Sports Medicine, MSH Medical School Hamburg, Am Kaiserkai 1, 20457 Hamburg, Germany; 35 G-Lab, Faculty of Applied Sport Sciences and Personality, BSP Business and Law School, Berlin, Germany

**Keywords:** Physical exercise, Sedentary behavior, Brain, Cognition, Dose

## Introduction

We thank Desgorces for taking the time to read our recent publication in the European Journal of Applied Physiology (Herold et al. 2025b) and providing an insightful commentary (Desgorces 2025). In his commentary, Desgorces (2025) supports our view that density is an important variable for analyzing and prescribing the dose and dosage of physical activity (PA) in the consideration of their effects on brain health. However, he also acknowledges that quantifying PA density is challenging, which is underlined by different perspectives on its definition, operationalization, and interpretation. In this context, we are pleased to have the opportunity for further clarification and for a constructive discussion by providing our perspective on the points raised by Desgorces (2025) in this reply. While we acknowledge the points raised in Desgorces’ commentary (2025), our response provides additional support for our understanding of PA density as conveyed in our initial review.

## Definition of density

Desgorces (2025) is accurate in noting that our definition of the term *density* differs from the International System of Units (i.e., Système International d’Unités; SI), which is an established and globally used measurement standard based on the metric system (Gilbey and Wilson 2020). Specifically, SI broadly defines density as *mass divided by volume*. In contrast, we provide a PA-specific definition of density, particularly as the *timing of PA (also referred to as work bout[s]) within a single PA bout as well as the timing between successive PA bouts within a specific time period (e.g., day, week, month, or year) by providing information on the temporal intervals (i.e., time spent resting; also referred to as rest, recovery, or relief bouts) within or between successive PA bouts* (Herold et al. 2025a, b; Manci et al. 2024).

Interestingly, a comparable discourse has emerged concerning the term (*training) load* (Impellizzeri et al. 2022a, b; Staunton et al. 2021, 2022). In this context, Impellizzeri and colleagues (2022a; b) assert that employing the SI to operationally describe and define a phenomenon in the broader field of exercise science (i.e., constructs such as *training load*, or exercise and training variables such as *density*) is not justified because the SI is neither an obligation nor a requirement for the purpose of scientific communication. Moreover, Desgroces’ (2025) narrow application for the definition of density does not acknowledge that the term density is often used in other scientific disciplines (e.g., psychology) to reflect the general principle relating to a quantity’s distribution (e.g., number of individuals; analogously number of PA and rest bouts) within a given space (e.g., per square mile; analogously total time spent on PA and timing of PA and rest bouts within this period) (https://dictionary.apa.org/density).

Thus, we believe that using the term *density*, when appropriately contextualized (e.g., as PA density), and as done by others (Bompa 2009; Hernández-Lougedo et al. 2021), is appropriate for scientific communication, although we acknowledge that others may prefer to use other terms, such as ‘*interbout interval*’ (Yin et al. 2025), to refer to this phenomenon.

## Operationalization of density

Desgorces’ commentary (2025) questioned the operationalization of *density* through the quantification of the duration of the rest/recovery bout(s)/interval(s)/period(s), as he assumes ‘*…that rest duration does not directly provide the value of density.*’ This appraisal is inconsistent with the definition and operationalization of density in the broader exercise science literature (Bompa 2009; Hernández-Lougedo et al. 2021). In direct support of this view, Bompa (2009) states, ‘*The density of training can be thought of as a relationship that is expressed in units of time between working and recovery phases of training. Thus, the greater the density of training, the shorter the recovery time between working phases of training.*’ Bompa’s definition (2009) and operationalization of density directly support our position (i) that this variable can be quantified by the rest duration between two successive bouts of PA, and (ii) that a greater PA density is characterized by shorter rest durations, and, vice versa, a lower PA density is defined by longer ones (Herold et al. 2025a, b; Manci et al. 2024). Thus, the alternative interpretation of PA density proposed in Desgorces’ commentary (2025), namely that ‘*a 3-min rest period should represent greater density than a 1-min period*’, should be treated cautiously because it is neither consistent with the general interpretation of this term in other scientific disciplines, such as psychology (e.g., Would one attribute ‘*greater density*’ to a non-crowded environment with more physical space per individual [analogously to a 3-min rest period], or to a crowed environment with less physical space per individual [analogous to a 1-min rest period]?—[https://dictionary.apa.org/density]), nor those provided in the broader exercise science literature (Bompa 2009).

Thus, and consistent with the broader exercise science literature concerning the definition and operationalization of density (Hernández-Lougedo et al. 2021; Bompa 2009), we maintain the opinion that (i) density can be quantified by the rest duration and timing of PA within a single PA bout as well as the timing between successive PA bouts within a specific time period, and (ii) a longer rest duration (e.g., more time between successive bouts of PA), which allows for a longer and tendentially more substantial recovery, reflects a *lower* instead of a *greater* density.

## Density in the context of physical activity dose and dosage

Desgorces (2025) states in his commentary that the manipulation of density would result in changes of the ‘*total exercise dose*’ (i.e., ‘…*when the protocols promoted changes in the exercise density, this concomitantly led to alterations in the total exercise dose.*’), and suggested an alternative approach to incorporate density to quantify PA dose (i.e., ‘*…we propose that the maximal duration achieved in each exercise design (including rest bouts) can represent the maximal dose (Desgorces et al. *2020*). The dose of an exercise would, thus, be defined as the percentage of this maximum.*’). Before discussing these points in more detail, it is imperative to clarify the terms *dose* and *dosage*, as a definition of ‘*total exercise dose*’ and an explanation of how it differs from ‘*maximal dose*’ are absent in Desgorces’ commentary (2025).

### Definition of dose and dosage in the broader physical activity literature

Within the exercise science literature, the *dose* is typically defined as a product of exercise variables (e.g., exercise intensity, exercise duration, type of exercise), and training variables (e.g., frequency of training sessions) (Solomon 2018; Wasfy and Baggish 2016; Williams et al. 2019). Therefore, dose is synonymous with *training load* (Impellizzeri et al. 2023), which is a higher order, multidimensional, and latent construct commonly defined as *input variable(s) to elicit a change in a specific outcome of interest* (Impellizzeri et al. 2019, 2020, 2023; McLaren et al. 2022). In this context, a single ‘gold standard’ metric for operationalizing the dose/dosage/training load is unlikely to exist, as the validity and appropriateness of a metric depend on the particular aim and application scenario of the intervention (Herold et al. 2019; Impellizzeri et al. 2023; Lambert and Borresen 2010; McLaren et al. 2022). For clarification and in reference to the comment by Desgorces (2025), in our previous work on this topic, we added the discourse on *dose* by proposing that (i) it can be defined as specific indicators of internal load for which there is evidence that they are (causally) involved in the complex psychophysiological processes that drive the desired changes in outcomes of interest (Gronwald et al. 2020; Herold et al. 2019, 2020, 2025b), with internal load as the psychophysiological response to external load (i.e., work performed by an individual independent of individual characteristics) and further influencing factors (e.g., environmental and/or personal factors) (Bourdon et al. 2017; Impellizzeri et al. 2019; Wallace et al. 2009), and (ii) it should be differentiated from the term *dosage* (i.e., dose provided over a particular time; Gronwald et al. 2020, Herold et al. 2020, Herold et al. 2025b)*.*

### Density—an essential piece of the puzzle to determine physical activity dose and dosage with higher precision

Although a definition of ‘*total exercise dose*’ is absent in the work of Desgorces (2025), it is plausible to assume that manipulating one exercise or training variable, regardless of whether this variable will be *frequency*, *intensity*, *time* (duration), *type*, or *density* of the PA intervention, will alter the *dose* and *dosage* of the intervention. Such an interpretation is well-supported by the scientific literature on this topic (Buchheit and Laursen 2013a, b; Impellizzeri et al. 2019, 2020, 2023; Toigo and Boutellier 2006). Undoubtedly, altering the PA dose and dosage by purposefully manipulating exposure to a particular exercise or training variable (i.e., density) is the key to isolating its effects on specific health-related outcomes (e.g., brain health), and thus for gaining a more comprehensive understanding of dose–response relationships that are often not well understood, especially in the PA-brain health field (Erickson et al. 2019, 2022).

As discussed in our previous work (Herold et al. 2025a, b; Manci et al. 2024), and reflected in relatively low reporting rates in empirical studies (see the systematic review with meta-analysis of Manser and colleagues (2024) for an example), PA density has been neglected in contemporary PA research practice compared to other exercise and training variables (e.g., exercise intensity and training duration), whose effects on health-related outcomes are frequently studied (e.g., in meta-analyses investigating PA effects on specific markers for brain health; Falck et al. 2019; Ludyga et al. 2020; Mavilidi et al. 2025; Northey et al. 2018; Singh et al. 2025). This is precisely why we call in our work for future research on density, a qualitative, yet underrecognized property of the PA dose and dosage, providing important information on the timing and clustering of PA (Herold et al. 2025a, b; Manci et al. 2024)—a view supported by Desgorces (2025).

As pointed out in our original work (Herold et al. 2025b), it is worth highlighting that manipulating the *density* should not be equated with manipulating *frequency* (i.e., the number of PA bouts within a specific time period (Herold et al. 2025a, b; Manci et al. 2024). Thus, when elucidating the effect of *density*, the total number of physical exercise sessions, as planned and structured forms of PA (Caspersen et al. 1985), within a specific time interval (i.e., frequency, such as three planned physical exercise sessions per week) should be kept comparable between groups exercising at different densities (e.g., the ‘low-density’ group exercises on Day 1, Day 3, and Day 5, whereas the ‘high-density’ group exercises on Day 1, Day 2, and Day 3; see also Fig. 1b in our original work; Herold et al. 2025b). Considering a time frame of 7 days provided in the example, it is unquestionable that a PA intervention that rigorously controls for frequency (i.e., with the same number of physical exercise bouts in both intervention groups) and other relevant exercise and training variables (e.g., intensity, type, duration) will result in different ‘final’ rest durations. In particular, the ‘high-density’ group will have a maximum of 4 days of rest after the last exercise session in a period of 7 days (i.e., Day 4 to Day 7) compared to the ‘low-density’ group which will have a maximum of 2 days of rest after the last exercise session in the same period (i.e., Day 6 and Day 7). While the pattern of the final rest durations between the two density groups differs fundamentally within the example period of 7 days, studies must be designed with careful consideration of the long-term periodization of the PA intervention (e.g., Does the ‘high-density’ group’s longer final rest duration at the end of the 7 days lead into another high-density exercise block in the next training period?). This inherently longer final rest duration in the ‘high-density’ group, when rigorously controlling for frequency, can be considered a potential challenge when studying the effects of density, depending on study aim and context, and is a consideration specifically demonstrated in Fig. 1b in our original work (Herold et al. 2025b).

Desgorces’ commentary (2025) posed the question of whether ‘*...density of exercise [would] remain unchanged when an exercise is performed at a higher intensity with an unchanged rest duration?*’. Based on the definition of density in our previous work (Herold et al. 2025a, b; Manci et al. 2024) and those provided in the broader exercise science literature (Bompa 2009; Hernández-Lougedo et al. 2021), PA density will remain unchanged in this example because the rest duration, as its key feature, is not manipulated. From a practical perspective, one could argue that the rest duration, and thus the density, could be adjusted (e.g., extended) to account for the increase in exercise intensity. However, such a potential adjustment of density strongly depends on further variables in the PA prescription puzzle, considering the intervention aims and context.

Taking the broader exercise science literature on PA dose and dosage (i.e., synonymous with training load) into account, we believe that the assumptions and contextualization of density in terms of the constructs provided in our original proposal (Herold et al. 2025b) are entirely valid and appropriate. However, as detailed in our previous work (Herold et al. 2025b), we agree with Desgorces’ position (2025) that a precise determination of PA dose and dosage, including the variable density, can indeed be ‘*tricky*’. This has been acknowledged in our previous work (Herold et al. 2025b) as follows: ‘*…we wish to emphasize that the precise characterization or prescription of a specific PA dosage will remain a considerable challenge…*’.

## Summary

In conclusion, we are grateful for the insightful commentary of Desgorces (2025), highlighting different views on the definition, operationalization, and interpretation of PA density, and providing an important impetus to discuss challenges related to the accurate determination of PA dose and dosage, particularly for PA density as one variable, in more depth. We hope that this reply and related discussion will contribute to a critical, yet constructive scientific discourse that will enable the identification of suitable approaches to determine PA dose and dosage, including PA density, in specific application scenarios with high precision. Characterizing the PA dose and dosage with high precision is an important prerequisite for a better understanding of PA-related health effects, which, in turn, is an essential piece of information for optimizing health-related PA recommendations and interventions.

## Data Availability

Data sharing does not apply to this article because no data were created or analyzed for this review article.

## Declarations

### Generative AI and AI-assisted technologies in the writing process

During the preparation of this work, the author(s) did not use generative AI or AI-assisted technologies. The author(s) take(s) full responsibility for the content of the published article.
